# Comparative Proteomics Demonstrates Altered Metabolism Pathways in Cotrimoxazole- Resistant and Amikacin-Resistant *Klebsiella pneumoniae* Isolates

**DOI:** 10.3389/fmicb.2021.773829

**Published:** 2021-11-18

**Authors:** Chunmei Shen, Ying Shen, Hui Zhang, Maosuo Xu, Leqi He, Jingbo Qie

**Affiliations:** ^1^Department of Hospital Infection Management, The Fifth People’s Hospital of Shanghai, Fudan University, Shanghai, China; ^2^Department of Clinical Laboratory Medicine, The Fifth People’s Hospital of Shanghai, Fudan University, Shanghai, China; ^3^Institute of Biomedical Sciences, Fudan University, Shanghai, China

**Keywords:** *Klebsiella pneumoniae (K. pneumoniae)*, comparative proteomics, bioinformatics, metabolism, antibiotic resistance

## Abstract

Antibiotic resistance (AMR) has always been a hot topic all over the world and its mechanisms are varied and complicated. Previous evidence revealed the metabolic slowdown in resistant bacteria, suggesting the important role of metabolism in antibiotic resistance. However, the molecular mechanism of reduced metabolism remains poorly understood, which inspires us to explore the global proteome change during antibiotic resistance. Here, the sensitive, cotrimoxazole-resistant, amikacin-resistant, and amikacin/cotrimoxazole -both-resistant KPN clinical isolates were collected and subjected to proteome analysis through liquid chromatography coupled with tandem mass spectrometry (LC–MS/MS). A deep coverage of 2,266 proteins were successfully identified and quantified in total, representing the most comprehensive protein quantification data by now. Further bioinformatic analysis showed down-regulation of tricarboxylic acid cycle (TCA) pathway and up-regulation of alcohol metabolic or glutathione metabolism processes, which may contribute to ROS clearance and cell survival, in drug-resistant isolates. These results indicated that metabolic pathway alteration was directly correlated with antibiotic resistance, which could promote the development of antibacterial drugs from “target” to “network.” Moreover, combined with minimum inhibitory concentration (MIC) of cotrimoxazole and amikacin on different KPN isolates, we identified nine proteins, including garK, uxaC, exuT, hpaB, fhuA, KPN_01492, fumA, hisC, and aroE, which might contribute mostly to the survival of KPN under drug pressure. In sum, our findings provided novel, non-antibiotic-based therapeutics against resistant KPN.

## Introduction

Antibiotic resistance (AMR) has always been a hot topic all over the world. The report released by World Health Organization (WHO) revealed that this serious threat is happening in every region of the world and has the potential to affect anyone, of any age, in any country (World Health Organization [WHO], 2014). In-depth research on the causes of bacterial drug resistance can provide effective guidance and support for us to fight against bacterial drug resistance.

Increasing evidences showed that metabolic changes in bacteria can reduce bacterial susceptibility to antibiotics and promote the evolvement of resistance, tolerance and persistence, and the regulation of physiological metabolism of bacteria can restore their sensitivity to antibiotics (Martinez and Rojo, 2011). Meylan et al. (2017) reported that metabolically dormant bacteria were genetically susceptible to antibiotic treatment, which could be reversed by adding glyoxylate into the culture. Mechanistic studies demonstrated that glyoxylate could induce phenotypic resistance of bacteria by inhibiting cellular respiration with acetyl-coenzyme A diversion through the glyoxylate shunt (Meylan et al., 2017). Another empirical study showed that aminoglycosides, such as glucose, mannitol, fructose, and pyruvic acid, improved the sensitivity of *Ataphylococcus aureus* and *Escherichia coli* to gentamicin by promoting glycolysis metabolic pathway (Allison et al., 2011). Barraud et al. (2013) also found that mannitol enhanced antibiotic sensitivity of persister bacteria in *Pseudomonas aeruginosa* biofilms. These findings were confirmed by the studies of other drug-resistant bacteria (Peng et al., 2015; Su et al., 2015, 2018; Koeva et al., 2017), suggesting that metabolic alteration have been a widely used strategy applied by bacteria to adapt the antibiotic pressure. Recently, protein aggresome was introduced into the study of AMR. Bai’ group found that the protein aggresome, whose formation is promoted by decreased cellular ATP level, was critical for AMR (Pu et al., 2019; Jing et al., 2020; Jin et al., 2021). These findings prove the important of metabolism in AMR, from another side. However, the specific metabolism-related protein profiles of resistant bacteria are still poorly understood.

Comparative proteomics has been extensively used to illustrate the dynamic changes of bacterial proteomes in the antibiotic stress (Freiberg et al., 2004). Resistant bacteria can revert its internal harmony which is disturbed by antimicrobials via modulating cellular protein expression and related pathways. By mapping the proteome of resistant bacteria, researchers could explain the observed experimental phenomenon and explore novel strategies employed by bacteria to gain antibiotic resistance. By now, proteomic study has been performed in *Escherichia coli*, *Bacillus subtilis*, and other micro-organisms (dos Santos et al., 2010; Hessling et al., 2013; Lata et al., 2015; Qayyum et al., 2016; Keasey et al., 2019).

*Klebsiella pneumoniae* (KPN) is a gram-negative pathogen, which was first identified in 1882 (Wang G. et al., 2020). In the 1960s, KPN have become one of the most important causes of opportunistic healthcare-associated infections (Paczosa and Mecsas, 2016). Nowadays, cotrimoxazole (CTX) and amikacin (AMI) are the antimicrobial of choice for treating CR-KPN (Ramirez and Tolmasky, 2017). But with the extensive use of cotrimoxazole and amikacin, even abuse, we find increasing cotrimoxazole-resistant, amikacin-resistant, even both-resistant KPN in our hospital, which is of great clinical concern. In this study, the sensitive, cotrimoxazole-resistant, amikacin-resistant, and amikacin/cotrimoxazole -both-resistant KPN clinical isolates in our hospital were collected and subjected to LC-MS/MS analysis. As a result, a deep coverage of 2,266 proteins were successfully identified and quantified in total, representing the most comprehensive protein quantification data by now. We applied comparative proteomics to explore the correlation between pattern characteristics and drug-resistances. The results showed absolute pattern identity between ATCC strains and hospital isolates, as well as altered energy metabolism processes between SEN isolates and drug-resistant isolates. Specifically, proteins involved in tricarboxylic acid cycle (TCA) pathway were down-regulated in both of amikacin- and cotrimoxazole -resistant KPNs, which may restraint the ROS production in drug-resistant isolates. Finally, we identified nine genes involved in metabolism pathways significantly associated with MICs of amikacin/cotrimoxazole, and participated in such an alteration. These results indicated that the alteration of metabolic network was directly correlated with antibiotic resistance, which could promote the development of antibacterial drugs from “target” to “network,” and provided novel, non-antibiotic-based therapeutics against resistant bacteria.

## Materials and Methods

### Strain Collection and Drug Susceptibility Testing

Three cotrimoxazole-resistant (CTX), three amikacin-resistant (AMI), three both-resistant (ACB), and three drug-sensitive (SEN) KPN isolates were collected by the microbiology lab at Shanghai Fifth People’s Hospital for this study. The KPN type strains (33259, 13883, and 11296) were purchased from American Type Culture Collection (ATCC, United States). Minimum inhibitory concentrations (MICs) were interpreted according to the Performance Standards for Antimicrobial Susceptibility Testing M100 edition 28 (2018) of the Clinical and Laboratory Standards Institute (CLSI) (Gehring et al., 2021).

### Culture Scaling and Sample Preparation for Proteome

For each KPN strain, three KPN monoclonals were inoculated in Luria-Bertani (LB) broth to the exponential phase (OD_600_ = 0.8), and KPN cells were collected and washed in PBS and subjected to global protein extraction using 8 M Urea (PH = 8.0) containing protease inhibitor, followed by 3 min of sonication (3 s on, 3 s off, amplitude 25%). Then the protein concentration was quantified through Bradford method and 100 μg protein was digested overnight following filter-acid sample preparation (FASP) method (Wisniewski et al., 2009) with 3.5 μg trypsin in 50 mM ammonium acid carbonate (PH 8.0) overnight at 37°C. Finally, the purified peptides were acquired after extraction with

50% acetonitrile (ACN) and 0.1% formic acid (FA) following desalination in two layers of Empore 3 M C18 disk with 2 mg packing (3 μm, 150 Å, Agela) in a pipet tip and dried in a vacuum concentrator (Thermo Fisher Scientific).

### Liquid Chromatography Coupled With Tandem Mass Spectrometry Analysis, Proteome Identification and Quantification With MaxQuant-Based Database Searching

Proteome analysis was processed on Q Exactive HFX mass spectrometer (Thermo Fisher Scientific, Rockford, IL, United States) coupled with Easy-nLC 1,000 nanoflow liquid chromatography system (Thermo Fisher Scientific). The MS raw files were searched against *Klebsiella pneumoniae* subsp. *Pneumoniae* (strain ATCC 700721) database (version 20171126) in the Uniprot Knowledgebase (UniProtKB) using Maxquant (Version 1.5.3.30) (Cox and Mann, 2008). Peptides (minimum length of seven amino acid residues) with 1% FDR and a Mascot ion score greater than 20 were selected for protein identification. Proteins with 1% FDR (with at least one unique peptide) were selected for further analysis. For the proteome quantification, the intensity-based absolute quantification (iBAQ) value was extracted from MaxQuant results and subjected to FOT calculation. FOT was defined as protein’s iBAQ divided by the total iBAQ of all identified proteins in one experiment. Finally, FOT was multiplied by 10^6^ for easy presentation. The geometric mean value of the copy numbers in three proteomic analyses of each macrophage population were calculated and used for protein quantification. The significantly different expressed proteins (DEPs) were filtered with the fold change > 2 and *P*-value < 0.05 (bilateral Student’s *t*-test).

### Hierarchical Clustering and Principal Component Analysis

Unsupervised hierarchical clustering was carried out using R package “pheatmap” (version 1.0.12). The distances between the rows or columns of a data matrix were computed based on the Euclidean distance. The “complete” method was used in agglomeration process. PCA was performed using R package “gmodels” (version 2.18.1) in the statistical analysis environment R version 4.0.0 based on the relative protein quantification values (FOT) of each sample.

### Gene Enrichment Analysis

Gene Ontology (GO) enrichment and Kyoto Encyclopedia of Genes and Genomes (KEGG) pathway analyses of the DEPs between sensitive and drug (AMI or CTX)-resistant isolates was performed with the R Bioconductor package “clusterProfiler” (version 4.0.2). Enrichment significance was determined using Fisher’s exact test.

## Results

### Label-Free Quantitative Proteome Identify the Pattern Characteristics of Hospital-Derived *Klebsiella pneumoniae* Strains

**FIGURE 1:**
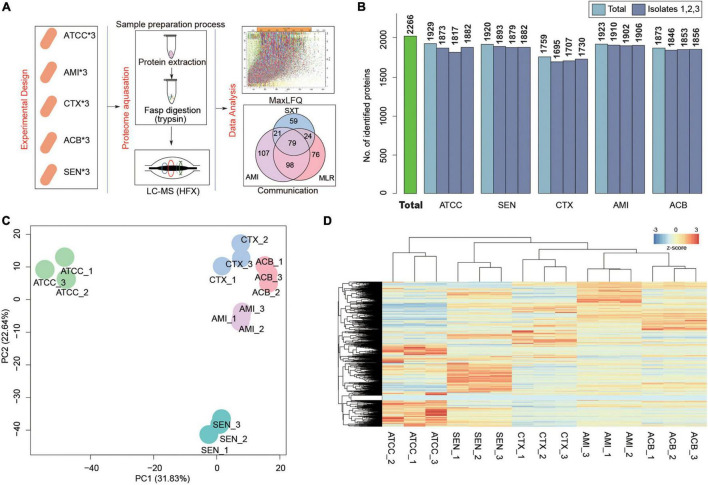
Label-Free Quantitative Proteome identify the pattern characteristics of hospital-derived KPN strains. **(A)** The experimental design and workflow. *Klebsiella pneumoniae* clinical isolates, including SEN, AMI-resistant, CTX-resistant, and ACB-resistant isolates, and ATCC strains were collected. The cells were subjected to LC-MS/MS analysis for label-free proteome following protein extraction and FASP digestion. Protein quantification were finished using Maxquant software with iBAQ algorithm. **(B)** The number of proteins detected in each isolate. **(C,D)** Principal component analysis **(C)** and Unsupervised hierarchical clustering **(D)** of the protein profiling of 15 KPN strains.

To search the global protein patterns of different KPN clinical isolates, a total of 15 KPN strains, including three sensitive (SEN), three amikacin-resistant (AMI), three cotrimoxazole-resistant (CTX), and three amikacin/cotrimoxazole both-drug-resistant (ACB) KPN isolates derived from 12 different patients (Table 1) in our hospital, as well as three ATCC type strains, were collected and subjected to LC-MS/MS in single runs by a quadrupole Orbitarp instrument after trypsin digestion for label-free proteomics analysis (Figure 1A). As a result, a deep coverage of 2,266 proteins were successfully identified and quantified in total (Figure 1B and Supplementary Table 1), expanding the experimental coverage of the 5,126 predicted bacterial gene products (in KPN database from UniprotKB, strain ATCC 700721) from 23% (1,156) (Sharma et al., 2019), 32% (1,654) (Keasey et al., 2019) to 44%, providing an extended characterization of KPN proteome. The dynamic ranges of protein quantification values in this study spanned over six orders of magnitude (Supplementary Figure 1A), with the most abundant proteins being rpsU, HupA, tufa, etc. (Supplementary Figure 1B). rpsU, also known as ribosomal protein S21 (rps21), was reported to contribute to fitness, stress-tolerance and host interaction (Koomen et al., 2018). HupA (heat-unstable α, HUα) is a subunit of a heterotypic dimer, Huαβ, resulting in high growth and a lowly pleiotropic phenotype (Abebe et al., 2017). tufA (translational elongation factor EF-Tu) was involved in a change in state or activity of a cell as a result of an antibiotic stimulus (Silk and Wu, 1993). The proteome quantification results of 15 experiments were comparable, as their medians were on the same level (Supplementary Figure 1C).

**TABLE 1:** Sample information.

Sample ID	Sample type	MIC_CTX (μ g/ml)	MIC_AMI (μ g/ml)
SEN1	Sputum	8	2
SEN2	Sputum	8	4
SEN3	Sputum	6	2
CTX1	Sputum	340	4
CTX2	Sputum	370	2
CTX3	Sputum	340	4
AMI1	Sputum	6	410
AMI2	Sputum	8	450
AMI3	Sputum	8	360
ACB1	Sputum	380	220
ACB2	Sputum	450	280
ACB3	Sputum	400	240

*SEN, drug-sensitive isolate; CTX, cotrimoxazole-resistant isolate; AMI, amikacin-resistant isolate; ACB, both-resistant isolate; MIC, minimum inhibitory concentration.*

To obtain the proteome identity of different strains systematically, PCA was carried out based on the protein abundance of KPNs. The result showed a very clear separation between ATCC and hospital isolates on the first component level as shown in *x*-axis, and captured the significant differences between sensitive and drug-resistant strains on the second component level as shown in *y*-axis (Figure 1C). An unsupervised hierarchical clustering analysis of the proteome patterns further supported the results (Figure 1D). Therefore, considering the fundamental differences between standard KPN strains from ATCC and clinical isolates from our hospital, we mainly focused on comparative analysis between proteome of three drug-resistant isolates (CTX, AMI, and ACB) and sensitive isolates for further study.

### Comparative Proteomics Analyses Revealed the Metabolism Alteration of Cotrimoxazole-Resistant Isolates Comparing With Sensitive Isolates

Cotrimoxazole is a combination of trimethoprim/sulfamethoxazole, and widely used for treatment of bacterial infections. The drug is extensively used due to the relatively cheap, available over-the-counter, and well tolerated (Morgan et al., 2011). Because of the injudicious use, the spread of bacteria with this antibiotic resistance has been a major factor (Okeke et al., 1999). Recent studies from the African continent have reported high rates of cotrimoxazole resistance in gram-negative pathogenic bacteria in the range of 50–96% (Manyahi et al., 2017).

**FIGURE 2:**
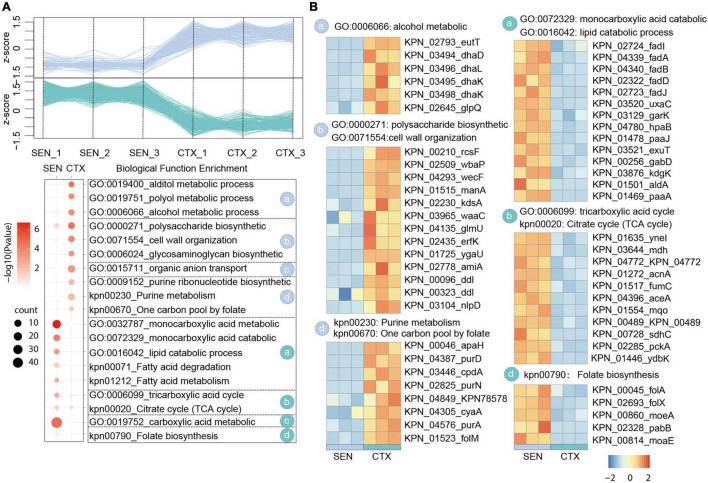
Comparative Proteomics Analyses Revealed the metabolism alteration of CTX-resistant isolates comparing with SEN isolates. **(A)** Expression profiles (up) and representative function enrichment analysis (down) of differentially expressed genes between CTX-resistant isolates and SEN isolates. Each line represents one protein. Functional terms were labeled and color-coded with *p*-value (Fisher’s exact test) according to the legend. **(B)** Expression patterns of proteins participating in the indicated cellular functions/pathways across CTX-resistant or SEN isolates. Values for each protein in all groups are color-coded based on the z-scored protein abundance per cell.

In our comparative proteome analysis between CTX-resistant isolates and SEN isolates, we identified 183 up-regulated proteins and 501 down-regulated proteins, respectively (Figure 2A and Supplementary Figure 2A). Further gene enrichment analysis showed an activation of alcohol metabolic process (eutT, dhaD, dhal) as well as a restraint of lipid catabolic process (fadl, fadA, fadB) and tricarboxylic acid (TCA) cycle pathway (ynel, mdh, acnA) in CTX-resistant isolates, which may contribute to ROS reduction and cell survival under drug pressure. It’s also worth noting that although the folate biosynthesis of cells (folA, folX, morA) was attenuated by CTX administration, the usage rate of one carbon unit was not affected accordingly illustrated by activation of purine ribonucleotide biosynthetic process and “one carbon pool by folate pathway (apaH, purD, cpdA). Besides, proteins in polysaccharide biosynthetic and cell wall organization processes (rcsF, wbaP, wecF), which may protect cell from drug perturbation through the physical safeguards” strategies, were up-regulated in CTX-resistant isolates (Figure 2B). There results showed the relative metabolism alteration of CTX-resistant isolates comparing with SEN isolates.

### Comparative Proteomics Analyses Revealed the Metabolism Alteration of Amikacin-Resistant Isolates Comparing With Sensitive Isolates

**FIGURE 3:**
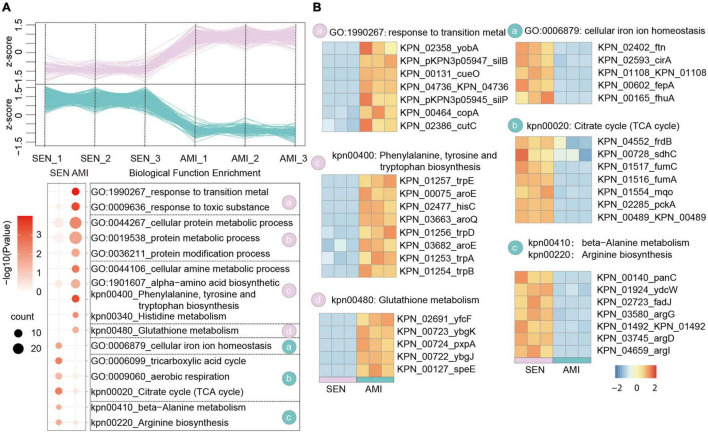
Comparative Proteomics Analyses Revealed the metabolism alteration of AMI-resistant isolates comparing with SEN isolates. **(A)** Expression profiles (up) and representative function enrichment analysis (down) of differentially expressed genes between AMI-resistant isolates and SEN isolates. Each line represents one protein. Functional terms were labeled and color-coded with *p*-value (Fisher’s exact test) according to the legend. **(B)** Expression patterns of proteins participating in the indicated cellular functions/pathways across AMI-resistant or SEN isolates. Values for each protein in all groups are color-coded based on the z-scored protein abundance per cell.

Amikacin shows a particularly broad antimicrobial activity which is used for severe bacterial infections. Nowadays, the emergency of amikacin-resistant KPN occurs dramatically with 20% in Turkey (Gokmen et al., 2016). Based on the proteome dataset, we identified 305 up-regulated proteins and 319 down-regulated proteins, respectively in AMI-resistant isolates comparing with SEN strains (Figure 3A and Supplementary Figure 2B). Gene enrichment analysis of these differentially expressed proteins indicated activation of glutathione metabolism pathway (yfcF, ybgK, pxpA) and restraint of TCA cycle pathway (frdB, sdhC, fumC), contributing to ROS clearance and cell survival under drug perturbation, in AMI-resistant isolates. Additionally, protein metabolic process as well as amino acid biosynthesis process was elevated in AMI-resistant isolates, indicating less affection of AMI administration in these isolates (Figure 3B).

Of note, combined with the results of CTX-resistant isolates, we found TCA cycle pathway was also inhibited in AMI-resistant isolates, and the downregulated proteins in TCA cycle pathway were high-degree overlapped, such as sdhC, fumC, mqo, pcka, and KPN_00489. These results reveal that restraint of TCA cycle pathway may be a widely used strategy of KPN to gain antibiotic resistance.

### The Expression Levels of Nine Metabolism-Associated Proteins Were Highly Correlated With the Degree of Drug-Resistance of *Klebsiella pneumoniae*

**FIGURE 4:**
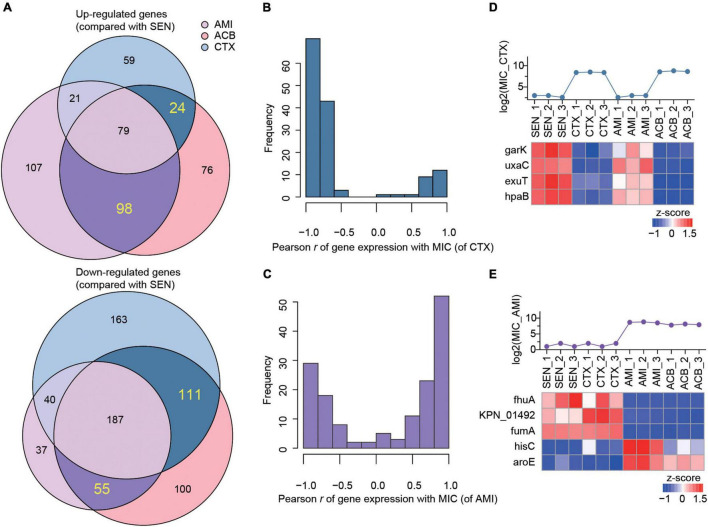
The expression levels of nine metabolism-associated proteins were highly correlated with the degree of drug-resistance of KPN. **(A)** Venn diagram summary of the number of up-regulated (up) or down-regulated (down) genes in AMI-resistant (purple), ACB-resistant (pink), and CTX-resistant (blue) isolates, comparing with SEN isolates. The genes only differentially expressed in one of AMI-resistant or CTX-resistant isolates were labeled with dark purple or dark blue, respectively. **(B,C)** The hist of Pearson correlation coefficients between expression patterns of genes. **(D,E)** Identification information of metabolic proteins whose expression patterns were significantly correlated with MIC of strains against CTX and AMI.

Combining with the proteome profiling of ACB-resistant isolates, we screened out a set of candidate proteins that were specifically associated with the mechanism of single antibiotic resistance of KPN. As a result, we identified 24 proteins whose expression were simultaneously up-regulated in CTX-resistant isolates and ACB-resistant isolates but not affected by AMI administration comparing with SEN strains, and 111 proteins on the contrary, as candidate proteins specifically for CTX-resistant isolates. Similarly, a total of 98 and 55 proteins exclusively up-regulated or down-regulated in AMI-resistant isolates, respectively, were identified as candidates specifically for AMI-resistant isolates (Figure 4A).

Next, to further identify the proteins closely correlated with mechanism of antibiotic resistance in KPN, we introduced the MICs of cotrimoxazole and amikacin on all the KPN hospital-isolates studied (Table 1). The Figures 4B,C showed the distribution of Pearson correlation coefficients between MICs and expression of protein sets above, respectively, for CTX-resistant isolates and AMI-resistant isolates. Finally, based on the results of gene enrichment analysis and correlation analysis, we identified nine proteins (Table 2), which were not only involved in the metabolic regulation of drug-resistant isolates, but also significantly correlated (positively or negatively) with the degree of drug resistance of KPN isolates (Figures 4D,E). Therefore, these enzymes possess important roles in reducing the ROS levels in cytoplasmic of cells. Further investigations need to be processed to determine the precise biological mechanism of the influence of these proteins on isolates resistance.

**TABLE 2:** Pearson correlation coefficients between MICs and expression of protein sets.

Gene_ID	Symbol*^a^*	Pearson *r^b^*	Function	FC*^c^*	*p* *^d^*
CTX group					
KPN_03129	garK	−0.9	Monocarboxylic acid catabolic process	0.30	4.72e-2
KPN_03520	uxaC	−0.98	Monocarboxylic acid catabolic process	0.35	1.11e-4
KPN_03521	exuT	−0.91	Monocarboxylic acid catabolic process	0.24	6.67e-4
KPN_04780	hpaB	−0.95	Monocarboxylic acid catabolic process	0.26	3.21e-3
AMI group					
KPN_00165	fhuA	−0.87	Cellular iron ion homeostasis	0.01	1.11e-3
KPN_01492	01492	−0.84	Arginine biosynthesis	0.48	5.20e-4
KPN_01516	fumA	−0.93	Citrate cycle	0.22	1.43e-4
KPN_02477	hisC	0.89	Phe, tyr, and try biosynthesis	48.18	1.16e-4
KPN_03682	aroE	0.95	Phe, tyr, and try biosynthesis	3.34	1.31e-2

*^a^Gene name from Uniprot.*

*^b^Pearson correlation coefficients between MICs and expression of protein.*

*^c^Fold change.*

*^d^P-value.*

## Discussion

Previous evidence has shown that bacteria can alter its metabolism to adapt the antibiotic pressure by reducing the accumulation of intracellular antibiotic (Richter et al., 2017). However, the specific metabolism-related protein profiles of drug-resistant bacteria are still largely understood (Liu et al., 2019).

In the present study, we found that proteins in TCA pathway were down-regulated in both CTX- and AMI-resistant KPNs, which may restraint the ROS production in drug-resistant isolates. The proteomics results showed the deficiencies of resistant KPN in central metabolic pathways (TCA cycle), compared with the sensitive KPN. Illuminated by the results from comparative proteomic approaches, our findings provide a mechanism to explain why adding alanine and/or glucose into culture could reduce drug resistance to the antibiotic (Bhargava and Collins, 2015; Peng et al., 2015; Su et al., 2015, 2018; Koeva et al., 2017). By re-analyzing the data reported previously, we found the deficiencies of resistant KPN in central metabolic pathways was a generally existent phenomenon. Briefly, the metabolic process, cellular process and response to stimulus were downregulated in ESBL-producing KPN (Wang Y. et al., 2020), and the Carbohydrate metabolic process, Generation of precursor metabolites and energy, Cellular amino acid metabolic process, Lipid metabolic process, Catabolic process, were all downregulated in carbapenem-resistant KPN (Sharma et al., 2019). These findings showed that restraint of TCA cycle pathway could be a widely used strategy of KPN to gain antibiotic resistance, showing a non-antibiotic based therapeutic method against antibiotic-resistant infections.

Besides the findings of TCA cycle and related pathways in resistant KPN, we also screened out nine resistance-related proteins participating in metabolic pathways, including garK, uxaC, exuT, hpaB, fhuA, KPN_01492, fumA, hisC, aroE, which participate in metabolism-related pathways and are associated with MIC of resistant KPN. garK play a role of glycerate kinase in phosphorylating glycerate to glycerate-2-phosphate, which is involved in the central metabolism of the cell (Wehrmann et al., 2020). uxaC, working as a D-glucoronate/D-galacturonate isomerase, is report to participate in catabolism of fructuronate (Utz et al., 2004). fumA encodes fumarase A (FumA), which participates in the tricarboxylic acid (TCA) cycle during both aerobic and anaerobic growth (Lin et al., 2012). Therefore, these enzymes possess important roles in the metabolism of KPN. Further investigations need to be processed to determine the precise biological mechanism of the influence of these proteins on isolates resistance.

In sum, proteomics was performed on the sensitive (SEN), cotrimoxazole (CTX)-resistant, amikacin (AMI)-resistant, and amikacin/cotrimoxazole-both (ACB)-resistant *Klebsiella pneumoniae* clinical isolates. A total of 2,266 proteins were identified and further bioinformatic analysis showed down-regulation of TCA pathway and up-regulation of alcohol metabolic or glutathione metabolism processes, which may contribute to ROS clearance and cell survival, in drug-resistant isolates. Finally, combined with MIC of amikacin and cotrimoxazole on different KPN isolates, we identified nine proteins, including garK, uxaC, exuT, hpaB, fhuA, KPN_01492, fumA, hisC, and aroE, that might contribute mostly to the survival of KPN under drug pressure, providing novel, non-antibiotic-based therapeutics against resistant KPN.

## Data Availability Statement

All the proteome data generated in this study, including the raw files, quantitative files, and final protein expression matrixes have been deposited to ProteomeXchange (http://www.proteomexchange.org) with accession number PXD028544.

## Ethics Statement

The studies involving human participants were reviewed and approved by the Ethics Committee of Shanghai Fifth People’s Hospital. The patients/participants provided their written informed consent to participate in this study.

## Author Contributions

JQ and LH conceived and supervised the study. JQ designed experiments and wrote the manuscript. CS performed experiments. CS, YS, HZ, and MX analyzed the data. All authors read and approved the manuscript and agreed to be accountable for all aspects of the research in ensuring that the accuracy or integrity of any part of the work are appropriately investigated and resolved.

## Conflict of Interest

The authors declare that the research was conducted in the absence of any commercial or financial relationships that could be construed as a potential conflict of interest.

## Publisher’s Note

All claims expressed in this article are solely those of the authors and do not necessarily represent those of their affiliated organizations, or those of the publisher, the editors and the reviewers. Any product that may be evaluated in this article, or claim that may be made by its manufacturer, is not guaranteed or endorsed by the publisher.

## Supplementary Material

Supplementary Figure 1 Overview of Label-Free Quantitative Proteome analysis. **(A)** The dynamic ranges of protein quantification values in this study spanned over six orders of magnitude. **(B)** The abundance of identified proteins with the most abundant proteins being rpsU, HupA, tufa, etc. **(C)** The proteome quantification results of 15 experiments.

Supplementary Figure 2 Volcano plot showing log2 fold change plotted against −log10 *P*-value. **(A)** For CTX-resistant isolates vs. SEN isolates. **(B)** For AMI-resistant isolates vs. SEN isolates. The red dots and green dots represent the upregulated and downregulated DEPs, respectively.
