# Supplementary material for: Comparative Proteomics Demonstrates Altered Metabolism Pathways in Cotrimoxazole- Resistant and Amikacin-Resistant Klebsiella pneumoniae Isolates
Source: Front Microbiol. 2021 Nov 18;12:773829. doi: 10.3389/fmicb.2021.773829 (PMC8637018; doi:10.3389/fmicb.2021.773829)
Supplement: Supplementary file 1 [file Data_Sheet_1.docx]

**Supplementary Material**

**LC–MS/MS Analysis**

Proteome analysis was processed on Q Exactive HFX mass spectrometer (Thermo Fisher Scientific, Rockford, IL, USA) coupled with Easy-nLC 1,000 nanoflow liquid chromatography system (Thermo Fisher Scientific). Peptide mixture were re-dissolved in solution A (0.1% FA) and loaded onto a C18 trap column (100 μm × 2 cm, homemade; particle size, 3 μm; pore size, 120 Å; SunChrom, USA) with a maximum pressure of 280 bar with solution A, then separated on a C18 separation column (150 μm × 12 cm, homemade; particle size, 1.9 μm; pore size, 120 Å; SunChrom, USA) with a gradient of 5-95% mobile phase B (80% ACN and 0.1% FA) at a flow rate of 600 nl/min for 75 min. The MS analysis was performed by scanning 300-1400 m/z with a resolution of 60,000 at 200 m/z, followed by up to 20 data-dependent MS/MS scanning with higher energy collision dissociation (HCD) at a normalized collision energy of 27% (max injection time 40 ms, isolation window 1.6m/z, target 2e3 ions), detected in the Orbitrap (R=15,000 at 200 m/z). Finally, the dynamic exclusion time was set at 18 s, and data were acquired by Xcalibur software (Thermo Fisher Scientific).

**Proteome identification and quantification with MaxQuant-based database searching**

MS raw files were searched against *Klebsiella pneumoniae* subsp. Pneumoniae (strain ATCC 700721) database (version 20171126) in the National Center for Biotechnology Information (NCBI) using Maxquant (Version 1.5.3.30) [21]. Trypsin was selected as the proteolytic enzyme, and two missed cleavages sites were allowed. The mass tolerance was 20 ppm for precursor and 0.5 Da for production. The oxidation of Methionine and N-acetylation were set as the variable modifications. The false discovery rates of the peptide-spectrum matches (PSMs) and proteins were set to less than 1%. Proteins with at least 1 unique peptide were selected for further analysis. For the proteome quantification, the intensity-based absolute quantification (iBAQ) value was extracted from MaxQuant results and subjected to FOT calculation. FOT was defined as protein’s iBAQ divided by the total iBAQ of all identified proteins in one experiment. Finally, FOT was multiplied by 10^6^ for easy presentation. The geometric mean value of the copy numbers in three proteomic analyses of each macrophage population were calculated and used for protein quantification. The significantly different expressed proteins (DEPs) were filtered with the fold change > 2 and P-value < 0.05.
