# Supplementary figures and images for: Comparative Proteomics Demonstrates Altered Metabolism Pathways in Cotrimoxazole- Resistant and Amikacin-Resistant Klebsiella pneumoniae Isolates
Source: Front Microbiol. 2021 Nov 18;12:773829. doi: 10.3389/fmicb.2021.773829 (PMC8637018; doi:10.3389/fmicb.2021.773829)

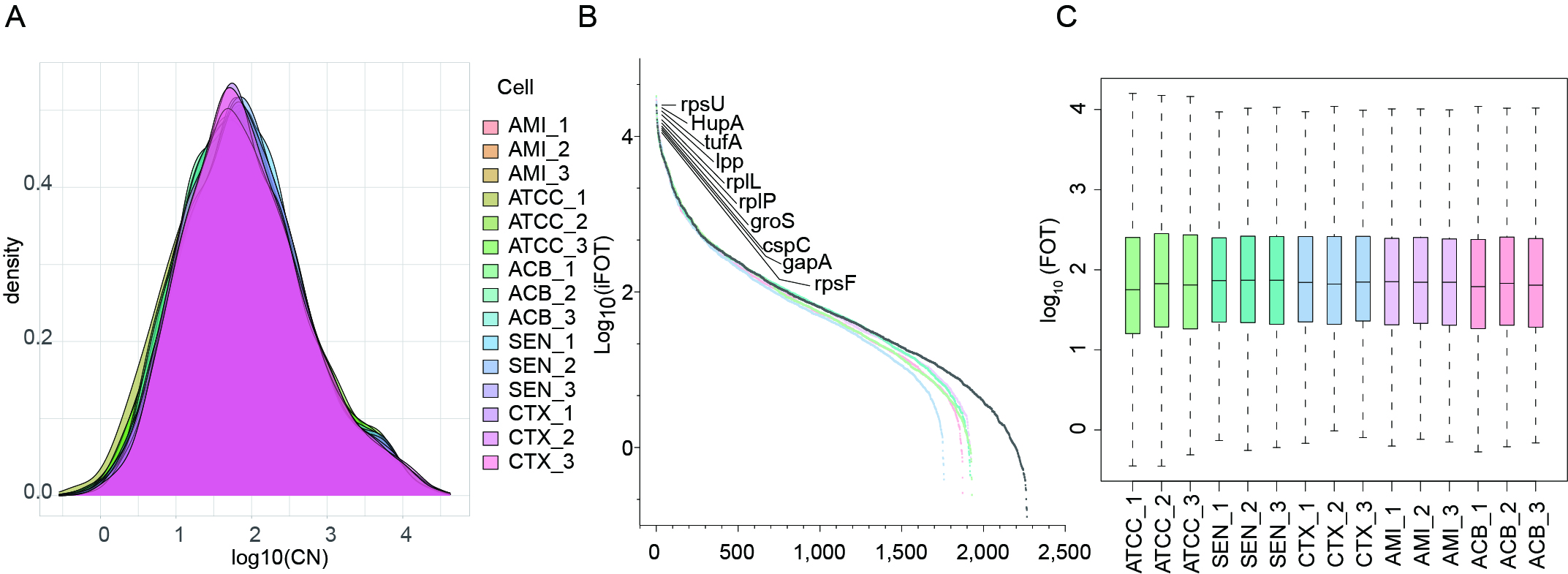

Supplement: Supplementary Figure 1 — Overview of Label-Free Quantitative Proteome analysis. (A) The dynamic ranges of protein quantification values in this study spanned over six orders of magnitude. (B) The abundance of identified proteins with the most abundant proteins being rpsU, HupA, tufa, etc. (C) The proteome quantification results of 15 experiments. [file Image_1.JPEG]

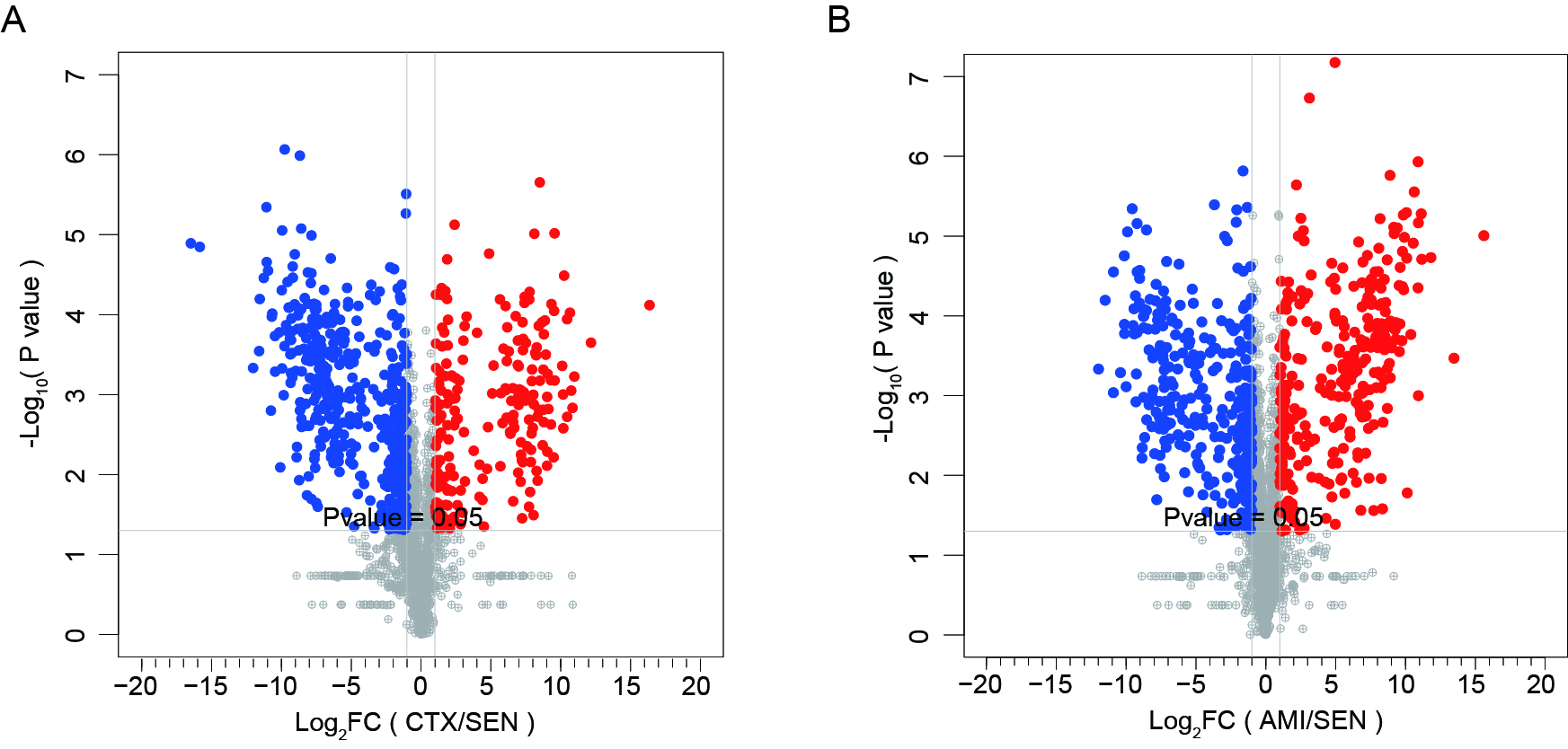

Supplement: Supplementary Figure 2 — Volcano plot showing log2 fold change plotted against −log10 P-value. (A) For CTX-resistant isolates vs. SEN isolates. (B) For AMI-resistant isolates vs. SEN isolates. The red dots and green dots represent the upregulated and downregulated DEPs, respectively. [file Image_2.JPEG]
